# The ADAMTS (A Disintegrin and Metalloproteinase with Thrombospondin motifs) family

**DOI:** 10.1186/s13059-015-0676-3

**Published:** 2015-05-30

**Authors:** Richard Kelwick, Ines Desanlis, Grant N Wheeler, Dylan R Edwards

**Affiliations:** School of Biological Sciences, Biomedical Research Centre, University of East Anglia, Norwich Research Park, Norwich, NR4 7TJ UK

## Abstract

The ADAMTS (A Disintegrin and Metalloproteinase with Thrombospondin motifs) enzymes are secreted, multi-domain matrix-associated zinc metalloendopeptidases that have diverse roles in tissue morphogenesis and patho-physiological remodeling, in inflammation and in vascular biology. The human family includes 19 members that can be sub-grouped on the basis of their known substrates, namely the aggrecanases or proteoglycanases (ADAMTS1, 4, 5, 8, 9, 15 and 20), the procollagen N-propeptidases (ADAMTS2, 3 and 14), the cartilage oligomeric matrix protein-cleaving enzymes (ADAMTS7 and 12), the von-Willebrand Factor proteinase (ADAMTS13) and a group of orphan enzymes (ADAMTS6, 10, 16, 17, 18 and 19). Control of the structure and function of the extracellular matrix (ECM) is a central theme of the biology of the ADAMTS, as exemplified by the actions of the procollagen-N-propeptidases in collagen fibril assembly and of the aggrecanases in the cleavage or modification of ECM proteoglycans. Defects in certain family members give rise to inherited genetic disorders, while the aberrant expression or function of others is associated with arthritis, cancer and cardiovascular disease. In particular, ADAMTS4 and 5 have emerged as therapeutic targets in arthritis. Multiple ADAMTSs from different sub-groupings exert either positive or negative effects on tumorigenesis and metastasis, with both metalloproteinase-dependent and -independent actions known to occur. The basic ADAMTS structure comprises a metalloproteinase catalytic domain and a carboxy-terminal ancillary domain, the latter determining substrate specificity and the localization of the protease and its interaction partners; ancillary domains probably also have independent biological functions. Focusing primarily on the aggrecanases and proteoglycanases, this review provides a perspective on the evolution of the ADAMTS family, their links with developmental and disease mechanisms, and key questions for the future.

## Gene organization and evolutionary history

Mammalian genomes contain 19 *ADAMTS* genes numbered 1 to 20, the designation *ADAMTS11* not being employed because it was assigned to a gene previously identified as *ADAMTS5* [1, 2]*.* Like their relatives, the matrix metalloproteinases (MMPs) and the ADAMs (A Disintegrin And Metalloproteinases), the ADAMTSs belong to the metzincin protease superfamily, named for the conserved methionine residue close to the zinc ion-dependent metalloproteinase active site [3]. Representatives of the ADAMTS family are found in all metazoans, although to date they have not been identified in single-cell organisms or in plants.

**Fig. 1:**
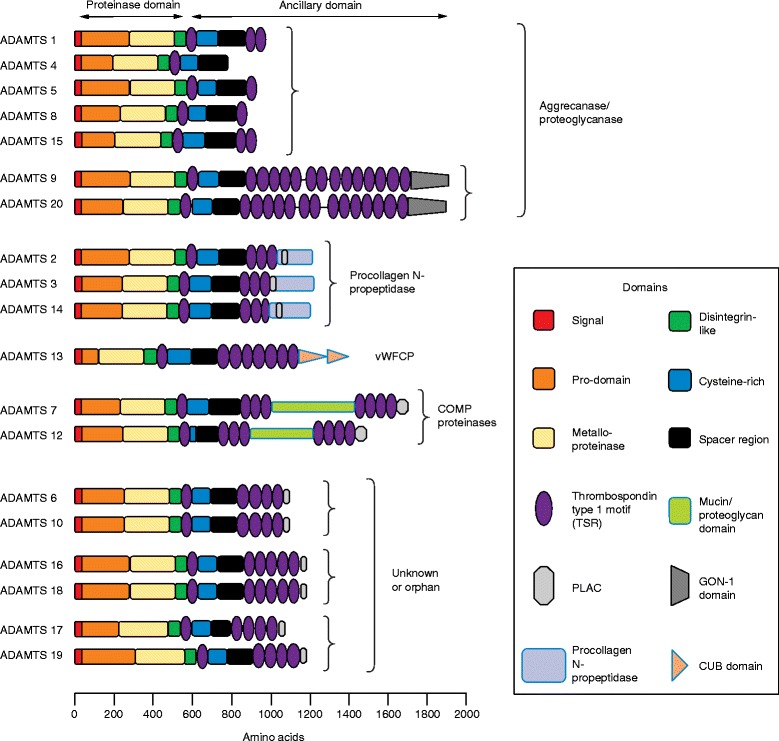
The ADAMTS (A Disintegrin And Metalloproteinase with ThromboSpondin motifs) family. The basic domain organization of the 19 ADAMTS family members and their major functional groups. Structurally the ADAMTS members are broadly organized into a proteinase domain and an ancillary domain. The proteinase domain comprises the signal, pro, metalloproteinase and disintegrin-like domains. The greatest variability between ADAMTS members is found in the ancillary domain, which is composed of one or more thrombospondin type 1 sequence repeats (TSRs), a cysteine-rich domain and a spacer domain. Some family members also have one or more specialist domains as part of their ancillary domain, as listed in the key on the right. The diagram is drawn to scale

All ADAMTSs are secreted, extracellular enzymes that have a compound domain organization (Fig. 1), comprising, from the amino-terminus: a signal peptide followed by a pro-region of variable length; a metalloproteinase domain; a disintegrin-like domain; a central thrombospondin type 1 sequence repeat (TSR) motif; and a cysteine-rich domain followed by a spacer region. This basic organization is manifest by ADAMTS4, and built upon in other family members with a variety of further carboxy-terminal modules, including one or more additional TSRs. The entire carboxy-terminal region downstream of the central TSR is termed the ancillary domain, and this is where the greatest differences between ADAMTS family members occur. Unlike their ADAM relatives, the ADAMTSs lack epidermal growth factor (EGF)-like, transmembrane and cytoplasmic modules. Separate from the ADAMTSs, another family of seven ADAMTS-like genes (*ADAMTSL*) encode proteins that resemble the ancillary domains of ADAMTS but lack their catalytic domains. These ADAMTSL proteins, which include ADAMTSL 1 to 6 and papilin, may function to modulate the activities of the ADAMTSs [2, 4].

The 19 human ADAMTS proteins can be assembled into eight ‘clades’ on the basis of their domain organization and their known functions. The aggrecanase and proteoglycanase clades (ADAMTS1, 4, 5, 8, 15, and ADAMTS9 and 20) can cleave hyaluronan-binding chondroitin sulfate proteoglycan (CSPG) extracellular proteins, including aggrecan, versican, brevican and neurocan [5]. This sub-group has also been labeled ‘angioinhibitory’ on the basis of the original identification of ADAMTS1 and 8 as anti-angiogenic factors [6]; nevertheless, ADAMTSs in other clades also have effects on angiogenesis. Another group (ADAMTS2, 3 and 14) are pro-collagen N-propeptidases that are essential for the maturation of triple helical collagen fibrils [7]. A lone family member, ADAMTS13, is the von-Willebrand factor (vWF)-cleaving protease (vWFCP). This protease processes large multimeric vWF precursor proteins under fluid shear stress conditions to generate vWF proteins of optimal size for proper blood coagulation [8]. Another clade (ADAMTS7 and 12) has been recognized as cleaving cartilage oligomeric matrix protein (also known as thrombospondin-5) [9, 10]. This clade is unique in the ADAMTS family in possessing a mucin domain to which chondroitin sulfate chains are attached, conferring proteoglycan status upon these two enzymes [11]. The remaining three subgroups, which are defined on the basis of their domain organizations, each contain a pair of enzymes (ADAMTS6 and 10; ADAMTS16 and 18; ADAMTS17 and 19) whose physiological substrates have yet to be identified, and thus are currently called the ‘orphan’ sub-groups. Like those ADAMTSs whose functions are better understood, several orphan enzymes have important physiological roles that are emerging from their associations with inherited human genetic disorders and acquired diseases.

**Fig. 2:**
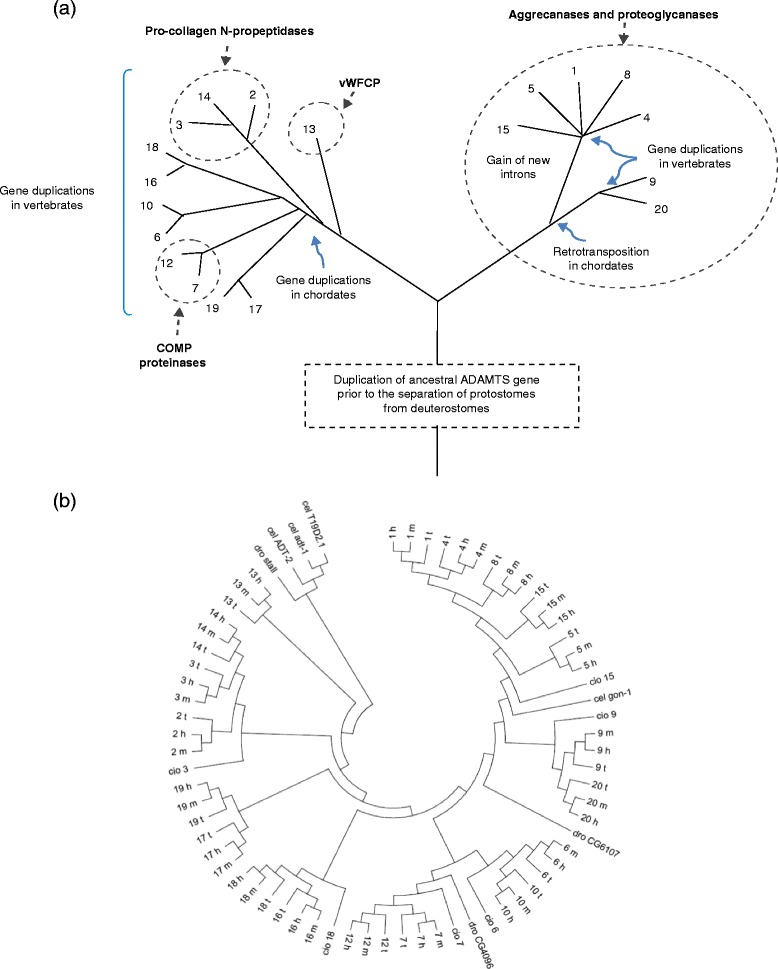
Evolution of the ADAMTS family. **a** A schematic representation of the relationships of the eight vertebrate ADAMTS clades and the probable events (gene duplications and a retrotransposition) that have contributed to the expansion of the family. The figure is not to scale in terms of evolutionary distance. COMP, cartilage oligomeric protein; vWFCP, von-Willebrand-factor-cleaving protease. **b** Phylogenetic tree of the *ADAMTS* genes inferred by the maximum likelihood method based on the JTT matrix-based model [144]. The bootstrap consensus tree inferred from 1,000 replicates was taken to represent the evolutionary history of the taxa analyzed [145]. Branches corresponding to partitions reproduced in less than 50 % bootstrap replicates were collapsed. Initial tree(s) for the heuristic search were obtained by applying the neighbor-joining method to a matrix of pairwise distances estimated using a JTT model. The analysis involved 70 amino acid sequences. All positions containing gaps and missing data were eliminated. Evolutionary analyses were conducted in MEGA6 [146]. The vertebrate *ADAMTS* genes are indicated by a number followed by a single letter code indicating the species: for example, 1 t represents 1_t *ADAMTS1* from *Xenopus tropicalis*; 15 h is 15_h *ADAMTS15* from *Homo sapiens*; 8 m is *ADAMTS8* from *Mus musculus*. For *Drosophila melanogaster* (dro), *Caenorhabditis elegans* (cel) and *Ciona intestinalis* (cio), the annotation is species followed by the gene number; for example, cio 6 is cio_6 *ADAMTS6* from *Ciona*

The pairs of ADAMTS proteins that share similar domain structures suggest the occurrence of gene duplication during evolution [12–16]. Comparison of the genomes of deuterostomes, namely those of the vertebrates *Homo*, *Mus* and *Xenopus* and that of the chordate *Ciona*, with those of protostome invertebrates (*Drosophila* and *Caenorhabditis*) has provided the view of the evolutionary history of the ADAMTS family summarized in the schematic and the phylogenetic tree in Fig. 2 [13, 16]. *Caenorhabditis elegans* and *Drosophila melanogaster* have four and three *ADAMTS* orthologs, respectively [12, 13]. Both have a single gene - *Gon-1* in the nematode and CG6107 in the fly - representing the right-hand branch of the human family shown in Fig. 2a; these genes are related to *ADAMTS9*/*20*. The left-hand branch is also represented by a single gene in *Drosophila* (CG4096) but it is missing entirely in *C. elegans*, which likely reflects loss of the founder gene during nematode evolution. Nevertheless, the remaining *Drosophila* gene (named *stall*) and the three *C. elegans ADAMTS-*related genes (*adt-1*, *adt-2* and *T19D2.1*) cluster as a protostome-specific sub-family that have no counterparts in deuterostomes [12].

Six *ADAMTS* genes are present in the basal chordate *Ciona intestinalis*, one of the closest invertebrate relatives of the vertebrates, each of which is the root of one of the eight mammalian clades (*ADAMTS3*, *6*, *7*, *9*, *15* and *18*), the two mammalian clades not represented in *Ciona* being *ADAMTS13* and the *ADAMTS17*/*19* pair [12]. This evidence argues that the gene pairs in vertebrates arose by duplication during their evolution from their chordate ancestors. The likely sequence of events is therefore that an ancestral *ADAMTS* gene duplicated approximately 650 million years ago, prior to the divergence of the protostomes (that is, the insects, crustaceans and nematodes) from the deuterostomes (chordates and vertebrates). One of these early duplicated genes gave rise ultimately to the aggrecanase/proteoglycanase sub-group (the right-hand branch in Fig. 2), while the other duplicate was the founder of the remaining family members. Subsequently, three or four gene duplications occurred during chordate evolution, with further duplications during vertebrate evolution.

The *ADAMTS9* and *20* gene pair in the aggrecanase/proteoglycanase sub-group share a high degree of similarity with the *C. elegans Gon-1* gene, suggesting their close relationship with the ancestral gene that founded the aggrecanases/proteoglycanases. On the basis of their intron and exon structure, however, the other related genes (*ADAMTS1*, *4*, *5*, *8*, *15*) seem likely to be derived from a retrotransposition event that occurred early in deuterostome evolution involving the same founder gene that also gave rise to *ADAMTS9*/*20.* This event seems to have produced an intron-less gene that subsequently acquired new introns at different positions. This evolutionary pathway is supported by the lack of orthologs of the *ADAMTS1*, *4*, *5*, *8*, *15* clade in protostomes [12–14]. Subsequently, this founder gene underwent a duplicative chromosome inversion to generate two adjacent *ADAMTS* genes in head-to-head orientation. A later duplication of this pair of genes resulted in two sets of paired genes, *ADAMTS1* and *ADAMTS5* on human 21q21, and the *ADAMTS8*/*15* pair on human11q24.

The deuterostome sea urchin *Strongylocentrus purpuratus* has eight *ADAMTS* orthologs that correspond to five of the eight ADAMTS clades present in vertebrates, but it lacks any representative for the *ADAMTS1*/*4*/*5*/*8*/*15* and *ADAMTS9*/*20* aggrecanase/proteoglycanase clades, suggesting that the progenitor of these genes has been lost [14]. This sea urchin has two genes with similarity to *ADAMTS13*, which had previously been thought to be a vertebrate-specific gene [12]. The presence of 16 *ADAMTS* genes in pufferfish (*Fugu rubripes*, which like *Ciona* lacks *ADAMTS13* and *17*/*19*), 17 genes in zebrafish (*Danio rerio*, which lacks *ADAMTS4* and *19*) and all 19 *ADAMTS* genes in *Xenopus* (*X. tropicalis* and *X. laevis*) argues that most of these gene duplications occurred prior to the divergence of fish and mammals. Among the zebrafish gene complement there are two copies each of *ADAMTS2*, *8* and *15*, which result from a teleost-specific whole-genome duplication event [16]. However, only 17 *ADAMTS* genes have been found in several species of birds: chicken (*Gallus gallus*), duck (*Anas platyrhynchos*) and zebra finch (*Taeniopygia guttata*) lack *ADAMTS4* and *16*, suggesting that these two genes were lost during the divergence of birds. The expansion of the *ADAMTS* family during vertebrate evolution goes hand-in-hand with the increased complexity of the ECM, which has also arisen through the duplication, retention and modification of ancestral genes [17].

## Characteristic structural features and mechanism

### Pro-domain

The pro-domains of the metzincins generally maintain latency and direct proper folding of the enzymes; but in the ADAMTSs they have additional functions. In general, ADAMTSs lack the ‘cysteine switch’ that controls activation of the MMPs, though curiously there is evidence for this in ADAMTS15. All ADAMTSs contain at least one site (R/KX_n_R/K↓R) for furin-like pro-protein convertases (PPCs) and some (such as pro-ADAMTS1 and −4) have been shown to be activated by PPCs in the *trans*-Golgi network, leading to secretion of active enzyme [18, 19]. Other ADAMTS precursors (such as pro-ADAMTS5) are activated by furin not in the Golgi but extracellularly [20], whereas pro-ADAMTS9 is activated on the cell surface when in a complex with the chaperone heat shock protein gp96/GRP94 [21]. Loss of the pro-domain actually reduces the activity of ADAMTS9 to cleave its substrate, versican; likewise pro-ADAMTS13 does not require pro-domain removal for catalytic activity [21–23]. The ADAMTS pro-domain may act to chaperone proper folding and secretion rather than in maintaining latency, although this is not the case for ADAMTS13. This enzyme has an unusually short pro-domain that probably acts by influencing binding to other proteins or by regulating catalytic activity in some other way [22].

### Catalytic domain: metalloproteinase and disintegrin-like modules

The ADAMTSs contain a consensus HEXXHXBG(/N/S)BXHD catalytic motif, in which the three histidines coordinate a Zn^2+^ ion [3]; B represents a large non-polar residue. A methionine, which in ADAMTSs lies 14 to 20 residues downstream of the third histidine, defines the ‘Met-turn’ common to the catalytic domains of all metzincin metalloproteinases [3]. In contrast to the ADAMs, in which 8 of the 21 human family members have lost a functional Zn-binding motif and are therefore proteolytically inactive [24], all ADAMTSs are predicted to be catalytically functional.

The ADAMTS metalloproteinase domains are predicted to adopt the typical metzincin architecture: a globular structure with an amino-terminal sub-domain comprising a five-stranded β-sheet on the top; and on the bottom, a carboxy-terminal sub-domain composed of α-helices in which the Met-turn is positioned, forming a hydrophobic pillow underneath the catalytic Zn^2+^. This creates an active site cleft into which substrates bind in essentially an extended, linear configuration [25]. A distinctive feature of ADAMTS metalloproteinase domains, when compared to those of the MMPs, is the presence of four disulphide bonds that stabilize the structure (MMPs have none) [26].

Unlike their ADAM relatives, no ADAMTS have been reported to interact with integrins via their disintegrin-like domains, and it has been suggested that this domain is misnamed in the ADAMTSs. Crystal structure data for ADAMTS1 [26] and for ADAMTS4 and 5 [27] reveal that the disintegrin-like domain is a cysteine-rich region that stacks against the metalloproteinase active-site cleft, so it is appropriate that we consider it part of the catalytic domain. A surprising finding from the crystal structures of the catalytic domains of ADAMTS4 and 5 is the identification of two alternative conformations of the active sites that may exist in equilibrium: an ‘open’ structure with an additional Ca^2+^ ion bound and a ‘closed’, inaccessible structure in which the Ca^2+^ ion is released [27]. The existence of two distinct conformational states has not been seen for MMPs or ADAMs (though crystal data are still sparse) and it may be an attribute of the ADAMTSs that is used to regulate their catalytic actions via binding accessory proteins, substrates or other ADAMTS domains.

### Ancillary domain

The ADAMTS enzymes rely upon their carboxy-terminal ancillary domains for their association with the ECM, for regulation of their activity, and for specification of their substrate-binding preferences. The ancillary domain in all ADAMTS enzymes contains an approximately 50-amino-acid thrombospondin-like repeat (TSR) that is similar to the type I repeats of thrombospondins 1 and 2 [28], followed by a cysteine-rich region of slightly more than 100 amino acid residues (in all except ADAMTS12) that contains 10 conserved cysteine residues, and finally, a more variable cysteine-free spacer region, which ranges from 103 to 160 amino acids in length. With the sole exception of ADAMTS4, the spacer domain is followed by 1 to 14 further TSR modules and additional motifs that are characteristic of particular subgroups. The ADAMTS9/20 pair has the largest number of TSRs and each concludes with a GON-1 module (first described in *C. elegans* Gon-1), which contains 10 conserved cysteine residues [23]. ADAMTS13 is unique in having two CUB modules. Several ADAMTSs (ADAMTS2, 3, 6, 7, 10, 12, 14, 16, 17, 18 and 19) possess a PLAC (protease and lacunin) module that contains six conserved cysteine residues. In ADAMTS7 and 12, a mucin/proteoglycan domain is interposed in the middle of the seven carboxy-terminal TSRs.

### Post-translational modifications

ADAMTS enzymes are glycosylated and their ancillary domains can be proteolytically processed, with both types of modification affecting the enzymes’ secretion, localization, activation or catalytic functions. All ADAMTSs with the exception of ADAMTS4 are N-glycosylated at NxS/T sites, with N-glycosylation within the pro-domain of ADAMTS9 being necessary for its secretion [21]. The TSRs are sites for *C*-mannosylation at tryptophans in conserved WxxW motifs, and *O*-fucosylation of serine or threonine residues in CxxS/TCG motifs, as demonstrated for the type I repeats in thrombospondins [29]. The *O*-fucosylation of ADAMTS13 regulates its secretion [30] and is probably a quality-control mechanism that ensures proper protein folding [2]. The mucin domain of ADAMTS7 is modified by the addition of CS GAG chains, which together with N-glycosylation of the pro-domain may regulate the enzyme’s cell-surface association and sequential processing by furin [11]. Proteolytic processing within the ancillary domains has been reported for many ADAMTS enzymes, and in some cases the cleavages are autolytic [18, 31, 32].

### Inhibitors of the ADAMTSs

Like the ADAMs, ADAMTSs show restricted susceptibility to inhibition by the four tissue inhibitors of metalloproteinases (TIMPs) [33]. Where multiple TIMPs have been tested, as is the case for ADAMTS2 [34] and ADAMTS4 [35], TIMP-3 emerges as the most effective inhibitor. The aggrecanase activity of ADAMTS1 is inhibited by both TIMP-2 and −3, but not by TIMP-1 and −4 [36]. The ancillary domains of ADAMTS4 and 5 promote interactions with TIMP-3 [37]. Inhibition of ADAMTS4 aggrecanase activity by TIMP-3 is enhanced by aggrecan, through a mechanism that involves the interaction of aggrecan GAG chains with the TSR and spacer regions of ADAMTS4 [38].

Another key difference between the ADAMTSs and the MMPs relates to their mode of inhibition by TIMPs. For MMP inhibition, the amino-terminal cysteine residue of a mature TIMP molecule is essential to coordinate with the active site Zn^2+^ [33]. Extension of TIMP-3 at the amino terminus by an alanine residue abrogates MMP inhibitory activity, but potent inhibition of ADAMTS4 and 5 is retained [39]. This may be significant in the design of selective ADAMTS inhibitors, such as *cis*-1(*S*)2(*R*)-amino-2-indanol-based compounds. These compounds are potent inhibitors of ADAMTS4 and 5, with K_*i*_ values in the low nM range and selectivity two orders of magnitude greater than that of the MMPs, suggesting that they are good platforms for the development of highly selective aggrecanase inhibitors [40]. Interference with substrate interactions by binding to exosites in the ancillary domains of ADAMTS is also a viable strategy, as shown by the use of calcium pentosan polysulfate for ADAMTS4 aggrecanase inhibition [41]. Also, granulin-epithelin precursor binds to the carboxy-terminal TSR motifs of ADAMTS7 and 12, blocking the abilities of these enzymes to cleave cartilage oligomeric protein (COMP) [42].

The activity of the ADAMTSs is also controlled by their internalization and degradation. For ADAMTS4 and 5 these processes have been shown to involve interaction with low-density lipoprotein-related protein-1 (LRP-1) [43, 44]. Differential affinity for LRP-1 results in different half-lives for extracellular ADAMTS4 and 5 [44]. As LRP-1 also binds and internalizes TIMP-3, and as this interaction is blocked by heparan sulfate GAGs, which also potentiates TIMP-3 activity, there is potential for modulating the tissue activities of ADAMTS using sulfated glycan mimics [45].

## Localization and function

The ADAMTS proteases have important roles in tissue development and maintenance, and their dysregulation or mutation is associated with a number of diseases. In the sections that follow we provide an overview of the current knowledge of the functions and regulation of ADAMTSs that has emerged from work on human pathologies and gene-knockout mice, concentrating on the aggrecanase/proteoglycanases. Other recent reviews have focused on the roles of ADAMTSs in arthritis [5, 46], cancer [47–51], atherosclerosis [52] and central nervous system injury and disorders [53].

Mutations in several *ADAMTS* genes are associated with human autosomal recessive Mendelian inherited disorders; this topic has been the focus of an excellent recent review [4]. The extreme skin fragility displayed by sufferers of Ehlers-Danlos syndrome type VIIC, which corresponds to dermatosparaxis in cattle and sheep, arises due to inactivation of *ADAMTS2* [41]. Recessive mutations in *ADAMTS13* are responsible for a condition called thrombotic thrombocytopenic purpura (TTP), which is caused by a failure to cleave the otherwise pro-thrombogenic ultra-large von Willebrand Factor multimers in the circulation, resulting in platelet aggregation and vessel occlusion [54]. Autoantibodies to ADAMTS13 also give rise to acquired TTP [55]. The connective tissue disorder Weill-Marchesani syndrome (WMS) manifests by short stature, brachydactyly, joint stiffness, cardiac valve stenosis and ectopia lentis (lens dislocation), and can be inherited in both autosomal dominant and recessive modes. Autosomal dominant WMS is attributable to mutations in the ECM protein fibrillin-1, which is required for formation of tissue microfibrils [56]. By contrast, autosomal recessive WMS is attributable to mutations in *ADAMTS10* [57]. ADAMTS10 binds to fibrillin-1 and −2 and promotes microfibril formation in the ECM [58], but it has been suggested that its function may be independent of its protease activity [4]. Significantly, mutations in two members of the *ADAMTSL* gene family (*ADAMTSL2* and *4*) result in syndromes whose phenotypes overlap the ocular, skeletal and cardiac features of WMS, and are also probably linked to proper microfibril formation in the affected tissues [4]. Likewise, mutations in *ADAMTS17* cause a recessive WMS-like phenotype [59]. Another inherited ocular syndrome, microcornea, myopic chorioretinal atrophy and telechanthus (MMCAT) is caused by mutations in the orphan gene *ADAMTS18* [60, 61]. Although not yet detected in human genetic disorders, inactivation of the *Adamts16* gene in rodents has been shown to lead to hypertension, cryptorchidism and male infertility, and aberrant renal development [62–64].

Table 1 summarizes the major tissue locations in which *ADAMTS* genes are expressed (see also the BioGPS database - http://biogps.org) and known ADAMTS substrates. Many *ADAMTS* genes are transcriptionally regulated by cytokines, growth factors, hormones and inflammatory mediators: major inducing and repressing stimuli or regulators are shown, though it must be emphasized that this list is not exhaustive. In Table 2 we have also listed the phenotypes of *Adamts* knockout animals, which are covered more extensively in another recent review [4].

**Table 1:** *ADAMTS* genes: their chromosomal positions, major tissue expression locations, expression-inducing factors, substrates, and associations with pathologies

Gene	Location	Expression	Factors inducing or (repressing) expression	Substrates	Pathology associations
***ADAMTS1***	21q21	Ovary, bronchial epithelial cells, fetal lung, placenta, smooth muscle, uterus, adrenal cortex, adipocyte, ciliary ganglion, prostate, olfactory bulb, breast stromal fibroblasts and myoepithelial cells	Progesterone, Brg1, IL-1, S100A8, S100A9, TNFα	Aggrecan, versican, syndecan 4, TFPI-2, semaphorin 3C, nidogen-1, −2, desmocollin-3, dystroglycan, mac-2, gelatin (denatured collagen type I), amphiregulin, TGF-α, heparin-binding EGF	Cancer (both pro- and anti-tumorigenic/metastatic), anti-angiogenic
***ADAMTS2***	5q35	Adipocyte, skeletal muscle, superior cervical ganglion, uterus, placenta, heart, liver, lung, tongue, smooth muscle, breast stromal fibroblasts	Glucocorticoids (in monocytes), IL-6	Fibrillar procollagens types I-III and V	Ehlers-Danlos syndrome type VIIc, dermatosparaxis (in sheep and cattle)
***ADAMTS3***	4q21	CD105+ endothelial cells, CD34+ cells, pineal gland, cartilage, bone, skeletal muscle, tendon, breast myoepithelial cells		Fibrillar procollagen type II, biglycan	
***ADAMTS4***	1q23	Ovary, spinal cord, adrenal cortex, ciliary ganglion, trigeminal ganglion, brain, retina, pancreas (islets), fetal lung, breast myoepithelial cells	IL-1 + oncostatin M, TNFα, S100A8, S100A9, leptin, IL-6	Aggrecan, versican, reelin, biglycan, brevican, matrilin-3, α2-macroglobulin, COMP	Arthritis
			(HDAC inhibitors, pentosan polysulfate)		
***ADAMTS5***	21q21	Adipocyte, uterus, breast myoepithelial cells	IL-1, TNFα, S100A8, S100A9, leptin, IL-6	Aggrecan, versican, reelin, biglycan, matrilin-4, brevican, α2-macroglobulin	Arthritis, cancer (anti-tumorigenic, anti-angiogenic)
			(HDAC inhibitors)		
***ADAMTS6***	5q12	Superior cervical ganglion, trigeminal ganglion, appendix, heart, breast myoepithelial cells	TNFα,	-	
***ADAMTS7***	5q24	Trigeminal ganglion, adrenal cortex, liver, heart, skeletal muscle, intervertebral disc, breast stromal fibroblasts	PTHrP	COMP	Coronary artery disease (smooth muscle cell migration)
			(miR-29a/b)		
***ADAMTS8***	11q24	Skeletal muscle, heart, liver, superior cervical ganglion, adrenal cortex, breast stromal fibroblasts and luminal epithelial cells		Aggrecan	
***ADAMTS9***	3p14	Dorsal root ganglion, breast myoepithelial cells	TNFα, IL1 + oncostatin M, leptin	Aggrecan, versican	Cancer (anti-angiogenic)
			(HDAC inhibitors)		
***ADAMTS10***	19p13	CD8+ T-cells, brain, uterus, breast stromal fibroblasts		Fibrillin-1	Weill-Marchesani syndrome
***ADAMTS12***	5p13	Liver, bone marrow, atrioventricular node, intervertebral disc, breast stromal fibroblasts and myoepithelial cells		COMP	Cancer (pro- and anti-tumorigenic)
***ADAMTS13***	9q34	Liver, CD71+ early erythroid cells, lung, thyroid, breast myoepithelial cells	(IL-1)	vWF	Thrombotic thrombocytopenic purpura
***ADAMTS14***	10q22	Thalamus, bone marrow, fetal thyroid, adipocyte, cerebellum, bone, skin, fibroblasts, breast myoepithelial and luminal epithelial cells		Fibrillar procollagen type I (pNα1 and pNα2 chains)	
***ADAMTS15***	11q24	Colon, brain, heart, uterus, musculoskeletal system, breast myoepithelial cells		Aggrecan, versican	Cancer (anti-tumorigenic/metastatic, anti-angiogenic)
***ADAMTS16***	5p15	Breast myoepithelial cells	Follicle stimulating hormone; forskolin (cAMP);	-	Hypertension
			Transcription factors: Wilm’s tumor-1; Egr-1, Sp1		
***ADAMTS17***	15q26	Breast myoepithelial cells		-	Weill-Marchesani-like syndrome
***ADAMTS18***	16q23	Ciliary ganglion, heart, skin, brain, breast myoepithelial cells		-	
***ADAMTS19***	5q23	Dorsal root ganglion, breast myoepithelial cells		-	
***ADAMTS20***	2q12	Brain, appendix, heart, liver, skeletal muscle, pituitary, trigeminal ganglion, breast myoepithelial cells		Versican

**Table 2:** ADAMTS knockout and mutant mouse phenotypes

Gene	Phenotype of gene knockout or mutant mice	Reference(s)
*Adamts1*	Growth retardation, adipose tissue malformation	[91]
	Impaired fertility with defective ovulation	[153]
	Severe kidney abnormalities: enlarged renal calices with fibrosis leading to obstruction of uteropelvic junction; abnormal adrenal medullary architecture with no formation of capillaries	[92] [148]
		[150]
	Defective follicular development during ovulation, delay in development of ovarian lymphatic vessels	[94, 95]
	Impaired skin wound healing; effects on keratinocyte and fibroblast migration	[98]
	No defects in aggrecan turnover *in vivo* or *in vitro*	[145]
	Reduced tumorigenesis and metastasis in PyMT mammary cancer, with increased apoptosis	[117]
	Defective myocardial morphogenesis	[96]
	Selective decline in synaptic protein levels in frontal cortex of female *Adamts1−/−* mice	[58]
*Adamts2*	Fragile skin at 1–2 months postnatal; male sterility	[144]
	Widespread defects in procollagen III processing; abnormal lungs	[142]
	Reduced extent and stability of carbon tetrachloride-induced hepatic fibrosis	[141]
*Adamts4*	No phenotype unchallenged	[69]
	Perinatal lethality, exacerbation of renal phenotype in *Adamts1−/−;Adamts4−/−* double knockout mice	[138]
*Adamts5*	No phenotype unchallenged. Protection in surgery-induced osteoarthritis and antigen-induced arthritis models	[69]
		[68]
	*Adamts4−/−;Adamts5−/−* double knockout mice phenotypically normal; osteoarthritis phenotype same as *Adamts5−/−* mice	[146]
	Blockade of fibrosis and accumulation of aggrecan in joints in the DMM and TTR models of osteoarthritis	[143]
		[139]
	Reduced changes in subchondral bone in DMM model of osteoarthritis	[152]
	Altered biomechanical properties of tendon	[81]
	Cardiac valve defects resembling myxomatous valve disease; rescued in versican (*Vcan*) heterozygotic animals	[147]
	Partial reduction of interdigital web regression	[151]
	Impaired dermal repair in excisional skin wound healing; aggrecan accumulation, altered **transforming growth factor** β (TGFβ) signaling	[140]
	Dermal fibroblasts have myofibroblastic phenotype showing increased contractility in three-dimensional collagen gels, rescued in versican (*Vcan*) heterozygotic animals	
*Adamts9*	Embryonic lethal at E7.5 days post coitum	[83]
	Partial reduction of interdigital web regression, enhanced in *Adamts5−/−;Adamts9−/+; bt/bt* mice	[82]
	Abnormal cardiac development in *Adamts9+/−* mice	[80]
*Adamts12*	No phenotype unchallenged; Elevated tumor growth and angiogenesis	[104]
	Exacerbated inflammation and airway dysfunction in allergen-induced airways disease	[149]
	More severe inflammation and delayed recovery following colitis, endotoxic sepsis and pancreatitis induction	[130]
*Adamts13*	Little phenotype unchallenged; loss of ADAMTS13 is pro-thrombotic but insufficient to generate thrombotic thromboscytopenic purpura	[137]
*Adamts20*	Mutations in *Adamts20* are found in *belted* (*bt*) mice, causing white spotting in the torso due to defective melanoblast survival	[83]
	Partial reduction of interdigital web regression, enhanced in *Adamts5−/−;Adamts9−/+; bt/bt* mice	[82]
	*Adamts9+/−;bt/bt* mice have cleft palate	[86]

### Development

A principal developmental role for the aggrecanases/proteoglycanases is in the cleavage of the CSPG versican. Versican is an essential ECM component during embryogenesis as it gives rise to a loose, hydrated hyaluronan-rich matrix that provides structural support while allowing dynamic remodeling during morphogenesis. It influences the adhesion, migration and proliferation of many cell types; versican-null mice die around E10 because of cardiac defects [65]. Versican cleavage is regulated during development to dismantle transitional structures, but versican cleavage fragments are themselves bioactive factors that modulate diverse cell signaling pathways [66]. Cleavage *in vivo* liberates the amino-terminal 70-kDa G1 hyaluronan-binding domain with a DPEAAE neo-epitope at its carboxyl terminus, which has been termed ‘versikine’ [67]. Antibodies against DPEAAE have shown that ADAMTS1, 4, 5, 9, 15 and 20 are versicanases in various contexts [68–70], including cardiac development [71, 72], limb morphogenesis [73], palate formation [74], skin pigmentation [75], and myogenesis [76, 77].

Developmental defects in versican cleavage underpin the phenotypes displayed by mice that are deficient in *Adamts1*, *5*, *9* and *20* (Table 2). During heart formation, the initially immature versican-rich ECM is replaced by a collagen, proteoglycan and elastin-containing matrix. *Adamts9*-null mice die prior to gastrulation, but hemizygous *Adamts9+/−* mice have heart malformations that reflect decreased detection of the DPEAAE neo-epitope and resulting accumulation of intact versican [71]. *Adamts5−/−* mice have enlarged heart valves by late fetal stages, correlating with reduced versican cleavage [72]. This phenotype was rescued by *in vivo* reduction of the level of versican by intercrossing with *Vcan* heterozygous mice. Thus, ADAMTS5 is required during heart development for the clearance of the early versican-rich matrix.

A similar requirement for removal of versican by ADAMTS5 is seen in skin development [78]. In this process, it is unnecessary to invoke a role for a neo-active versikine as partial depletion of versican restored normality. A dramatically different outcome is seen, however, in other morphogenetic events, including inter-digital web regression during autopod development, failure of which results in syndactyly, or webbing of the fingers and toes. Mice carrying combinations of null alleles for Adamts5, Adamts9 and Adamts20 (the latter gene is also known as the ‘belted’ locus, bt) show a failure of web regression, along with reduced versican cleavage and apoptosis [73]. Thus, the combined proteolytic activities of ADAMTS5, 9 and 20 are required to keep versican proteolysis above a threshold required for web regression. The strategy of reducing *in vivo* versican levels by heterozygosity resulted in 100 % penetrant syndactyly when combined with the absence of a single protease, ADAMTS20. In this context, therefore, the bioactivity of a versican cleavage fragment is probably required to promote web regression, a hypothesis that was confirmed by administration of recombinant G1-DPEAAE versikine. A similar outcome was observed with regard to palate formation [74]. Cleft palate occurs as a result of the failure of the two lateral palatal epithelial shelves to fuse and undergo epithelial-mesenchymal transition. *Adamts9+/−;bt*/*bt* mice show fully penetrant cleft palate, which is associated with decreased versican cleavage. Once again, versican heterozygosity exacerbated the phenotype seen with mice deficient in ADAMTS20 alone, suggesting that ADAMTS9 and 20 co-operate in the fusing palate. Functional co-operation of ADAMTS9 and 20 is also seen in their roles in melanoblast survival linked to coat pigmentation [75].

Versican cleavage by ADAMTS1 also underlies the roles of this enzyme in ovulation and cardiac development. *Adamts1-*null mice have extensive perinatal lethality, with surviving animals showing decreased growth and abnormalities in ureteral, adrenal and adipose tissues and infertility in female mice, indicating the importance of this enzyme in organogenesis and ovulation [79, 80]. ADAMTS1 expression by the granulosa cells of ovarian follicles is induced by progesterone, leading to follicle rupture by cleavage of the surrounding versican-rich matrix, which allows release of the oocyte [81, 82]. In *Adamts1−/−* mice, both ovulation and subsequent fertilization were severely impaired as a result of the persistence of versican. In cardiac development in mice, *Adamts1* expression is repressed by a chromatin remodeling protein, Brg1, leading to an extracellular environment within the cardiac jelly that supports the formation of the myocardial trabeculae [83]. Subsequently, ADAMTS1 production is switched on to terminate trabeculation, which coincides with the disappearance of versican. The participation of several ADAMTS enzymes in cardiac development suggests that they may also be involved in cardiomyopathies and heart failure in humans.

### Cardiovascular disease

In addition to the roles of versican in development, there is growing - and conflicting - evidence that this proteoglycan and its cleavage by ADAMTS enzymes may be associated with vascular pathologies [52]. It is present in atherosclerotic intimal thickenings and in advanced lesions, where it contributes to lipid retention and may influence the adhesion and recruitment of macrophages. In atherosclerotic lesions, ADAMTS1 is expressed by smooth muscle cells, whereas ADAMTS4 is a product of macrophages and its levels increase during lesion development [84]. Versican cleavage by ADAMTS1 could promote atherosclerosis through the activity of versikine in stimulating the migration of vascular smooth muscle cells (VSMCs) [85]. By contrast, ADAMTS5 has been suggested to be protective as it is depleted in atherosclerotic aortas, leading to accumulation of versican and biglycan [86]. ADAMTS7 is also linked to vascular disease because it promotes VSMC migration mediated by cleavage of COMP [87, 88]. Genome-wide association studies have identified *ADAMTS16* as a candidate locus involved in inherited hypertension [89], and this candidacy is supported by targeted disruption of *Adamts16* in a rat model [90].

### Arthritis

The ‘aggrecanase’ moniker for the aggrecanase/proteoglycanase group of enzymes originated in the arthritis field. Osteoarthritis (OA) emerges as a result of a progressive loss of aggrecan from cartilage, leading to exposure of the collagen matrix and its breakdown by MMP13 [91]. The most significant aggrecan cleavage site for OA pathogenesis is located at a highly conserved sequence TEGE^373^↓^374^ARGS, at which MMPs do not cut. Antibodies that recognize the ^374^ARGS neo-epitope led to the discovery of aggrecanase-1, which proved to be ADAMTS4 [92] and aggrecanase-2, which is ADAMTS5 [93]. Subsequently, other related ADAMTS enzymes, including ADAMTS1, 8, 9 and 15, were shown to have aggrecanase activity [28, 36, 94–96]. ADAMTS16 and 18 are also weak aggrecanases [97].

Various lines of evidence indicate that ADAMTS4 and 5 are the principal enzymes involved in the pathogenesis of the arthritides [91, 98]. They are the major aggrecanases present in cartilage, though *in vitro* ADAMTS5 is about 1,000 times more potent than ADAMTS4 [99]. In human cartilage explants and chondrocytes, knockdown of either ADAMTS4 or 5 (but not ADAMTS1) attenuated aggrecan breakdown [100], suggesting that both enzymes may be involved in human tissues. Expression of these two enzymes is augmented by cytokines such as interleukin-1 and oncostatin-M, which provoke aggrecan breakdown in tissues [101]. In mice, however, ADAMTS5 alone is the critical enzyme, as *Adamts5−/−* mice show significantly reduced joint destruction when compared to wild-type or *Adamts4−/−* mice in surgical and allergen-induced models of arthritis (Table 2) [102, 103]. Other ADAMTSs may be physiologically relevant aggrecanases in tissues other than cartilage: for example, aggrecan cleavage co-localized with ADAMTS1 in the developing kidney and was reduced in *Adamts1−/−* embryos when compared to wild-type animals. Local cofactors such as Fibulin-1, which binds to ADAMTS1 and increases its aggrecanase activity, may be important in determining which enzyme has the principal activity in a particular tissue context [104]. Also, ancillary domain cleavages change enzyme activities: autolytic and MMP17-mediated processing of ADAMTS4 in its ancillary domain enhance its ability to cleave aggrecan [105, 106]. Thus, ADAMTS5 (and in humans ADAMTS4) is a target for therapeutic inhibition. Selective inhibitors are yielding promise, as shown by the protective effects of the aggrecanase-selective inhibitor AGG-523 in a rat joint-instability-induced model of arthritis [107].

## Cancer

Numerous *ADAMTS* genes have been linked to cancer development and progression, with both promoting and antagonistic actions apparent. These dual roles probably reflect the effects of ADAMTS enzymes on the tumor microenvironment, affecting the interplay between malignant cells, the local stroma and the immune system. For many family members, the dominant theme is tumor suppression as they show epigenetic silencing (*ADAMTS1*, *8*, *9*, *12* and *18*) or mutational inactivation (*ADAMTS15*) in several cancer types [48, 51, 108–114]. *ADAMTS1*, *3*, *5*, *8*, *9*, *10* and *18* are down-regulated in human breast carcinomas compared to normal tissue, while only *ADAMTS4*, *6*, *14* and *20* are up-regulated [115].

Though originally reported for ADAMTS1 and 8, many ADAMTSs have proved to be anti-angiogenic, a property that may contribute to their tumor-suppressive actions [6, 116–121]. Metalloproteinase-dependent and -independent activities have been observed, with ADAMTS1 displaying both types of mechanism [122]. This enzyme cleaves matrix-bound thrombospondins-1 and −2, generating bioactive anti-angiogenic fragments [123], and also sequesters angiogenic factors such as the vascular endothelial growth factor VEGF_165_ [124] and basic fibroblast growth factor [125]. Metalloproteinase-independent inhibition of neovascularization has also been seen for ADAMTS2 [116], ADAMTS4 [118], ADAMTS5 [120] and ADAMTS12 [117]. The TSR domain may be responsible for these protease-independent activities since peptides (termed ‘Adamtsostatins’) corresponding to the TSRs of several family members are anti-angiogenic, indicating that this may be a general activity of the ADAMTSs [121, 126]. Metalloproteinase-dependent anti-angiogenic activity is displayed by ADAMTS9, but the mechanism is as yet unknown as thrombospondin cleavage has been ruled out [119]. The identities of the cryptic angio-inhibitory substrates of ADAMTS9 and ADAMTS15 [96] will be important to elucidate.

That ADAMTSs can have both pro- and anti-tumorigenic functions, depending on the cancer and its context, is best illustrated by ADAMTS1, which is down-regulated in breast, primary head and neck carcinomas, and epigenetically silenced in around 85 % of colorectal cancer cell lines [115, 127, 128]. Nevertheless, it appears to promote metastasis where its expression is activated later in tumor progression [122, 127, 129]. Its tumor-promoting action dominates in the highly aggressive MMTV-PyMT mouse mammary cancer model: *Adamts1−/−;PyMT* mice show reduced tumor growth and metastasis as a result of increased tumor cell apoptosis, while the cell proliferation and vascularity of their tumors were unaffected [130]. Tumors in mice displayed reduced cleaved versican and increased presence of cytotoxic immune cells, suggesting that ADAMTS1 creates a tumor-promoting microenvironment in the mammary gland. This may involve the shedding of epidermal growth factor receptor (EGFR) ligands, which has been linked to promotion of metastasis in other syngeneic mouse models [131] and to the collaboration of ADAMTS1 with MMP-1 to promote bone metastasis of human xenografts [132]. Other mechanisms that potentially contribute to the metastasis-enhancing effects of ADAMTS1 include promotion of cell migration by cleavage of Syndecan 4 and Semaphorin 3C [133, 134], and induction of endothelial mimicry by melanoma and sarcoma cells [135].

Tumor-suppressive effects of the ADAMTSs have been linked to the deactivation of key proliferation or survival signaling pathways, including suppression of Erk signaling by ADAMTS8 [136], ADAMTS12 [137] and ADAMTS15 [113], and of Akt/mTOR activity by ADAMTS9 [138]. Recently, ADAMTS15 was shown to inhibit breast cancer cell migration independently of its catalytic activity by increasing cell surface syndecan-4 levels [96].

## Frontiers

We are still short of understanding the functions of the ADAMTS family in development and disease. This truth is patent for the orphan enzymes for which substrates have yet to be discovered. Even for those ADAMTS (such as the aggrecanases) for which we have some knowledge of (some of) their substrates and the importance of their cleavage, it is likely that we will find new specialized roles in particular tissue contexts linked to the proteolysis of novel targets. There is a need, therefore, to apply proteomics technologies to unravel the ADAMTS substrate degradomes, working at the levels of both cells and intact tissues [139]. Building on this base, the identification of extracellular bioactive fragments that are generated by ADAMTSs’ action and investigation of the functions of these fragments will be priorities, along with determining the ways in which cofactors such as the fibulins [104, 140] influence binding and cleavage preferences.

Gene knockout mice have determined the developmental roles of certain ADAMTS family members, though coverage is incomplete [4]. The early lethality in *Adamts9−/−* mice indicates an essential developmental function for ADAMTS9 but restricts investigations to the study of heterozygous animals. Now a new conditional *Adamts9* model has been generated and a limb-specific knockout has confirmed an essential role for ADAMTS9 in interdigital web regression [141]. The generation of conditional knockout strains for other *Adamts* genes to chart their involvement in morphogenesis and disease will be essential.

At the protein level, there are many gaps in our knowledge of ADAMTS structure. In particular, we need to know how features within the pro- and ancillary domains of these enzymes conspire with the distinctive attributes of the ADAMTS catalytic domains to control enzyme localization, activation, trafficking, substrate binding, cleavage, inhibition and turnover. This knowledge will also prove useful in the design of novel therapeutics. Active-site-directed inhibitors that are selective for ADAMTSs over other metalloproteinases are a reality, but it may be difficult to build in an ability to discriminate between ADAMTS family members. Thus, although ADAMTS4 and ADAMTS5 inhibitors could be useful anti-arthritics, they may have unwanted toxicities if they also hit the pro-collagen-N-proteinases, for example. We would also need to know whether such agents have longer-term consequences, for instance in vascular function and angiogenesis. There is, however, potential to build on knowledge of exosite interactions to develop novel inhibitors that may block cleavage of specific substrates, while leaving other catalytic actions of the targeted enzyme unaltered.

Finally, an area that has not been covered extensively in this review is the regulation of *ADAMTS* expression, which is important for understanding disease pathogenesis. There is also the possibility of prevention of disease, potentially through dietary consumption of protective phytochemicals that down-tune *ADAMTS* expression [142, 143]. At the post-transcriptional level, our understanding of the functions of small RNAs as master regulators of gene systems that include the *ADAMTSs* is now advancing rapidly. Finally, given the roles of *ADAMTSs* in human diseases, it is likely that genetic polymorphisms that affect either their transcriptional or post-transcriptional control will be revealed.

## Abbreviations

ADAM: A Disintegrin and Metalloproteinases
ADAMTS: A Disintegrin and Metalloproteinase with Thrombospondin motifs
*ADAMTSL*: ADAMTS-like gene
COMP: Cartilage oligomeric protein
CSPG: Chondroitin sulfate proteoglycan
ECM: Extracellular matrix
EGF: Epidermal growth factor
LRP-1: Low-density lipoprotein-related protein-1
MMP: Matrix metalloproteinase
OA: Osteoarthritis
PPC: Pro-protein convertase
TIMP: Tissue inhibitors of metalloproteinases
TSR: Thrombospondin type 1 sequence repeat
TTP: Thrombotic thrombocytopenic purpura
VSMC: Vascular smooth muscle cell
vWF: von-Willebrand factor
vWFCP: von-Willebrand-factor-cleaving protease
WMS: Weill-Marchesani syndrome
